# The effects of surgical treatment with chondroblastoma in children and adolescents in open epiphyseal plate of long bones

**DOI:** 10.1186/s12957-018-1314-9

**Published:** 2018-01-23

**Authors:** Yan Xiong, Yun Lang, Zeping Yu, Hongyuan Liu, Xiang Fang, Chongqi Tu, Hong Duan

**Affiliations:** Department of Orthopedics, West China Hospital, Sichuan University, No 37 Guo Xue Lane, Wuhou District, 610041 Chengdu, Sichuan People’s Republic of China

**Keywords:** Chondroblastoma, Epiphysis, Open epiphyseal plate, Limb-length, Children and adolescents

## Abstract

**Background:**

Chondroblastoma is a rare benign cartilaginous tumor, which primarily occurs in children and adolescents. Chondroblastoma commonly originates in the epiphyseal plate of long bones. An aggressive curettage treatment is recommended to manage lesion, which may jeopardize an open epiphyseal plate and result in limb shortening and deformity as the limb grows and develops. The purpose is to observe surgical effects of chondroblastoma on open epiphyseal plate of long bones in children and adolescents and explore influences on limb growth and development.

**Methods:**

We retrospectively reviewed 18 cases of long bone chondroblastoma with open epiphyseal growth plate during March 2004 to October 2010 in our center. Seven females and 11 males with mean age of 11.6 ± 2.0 years old (8–15 years) were included. Patients, who suffered from trauma and pathological fracture of the epiphyseal plate or congenital diseases such as poliomyelitis, congenital dementia, and cartilage malnutrition, were excluded. All patients were treated with meticulous intralesional curettage and inactivity with alcohol followed by bone grafts. All cases were followed up 8.2 ± 1.7 years (5–11.5 years).

**Results:**

All had no local recurrence and distance metastasis. The length of the affected limb was short, 18.47 ± 7.22 mm (1.5–30 mm). There was no obvious relativity with tumor activity (*P* = 0.061). Meanwhile, there were obvious relativity with the greatest dimension of the lesion (TGD) (*P* = 0.003), the vertical dimension between edge of lesion and epiphyseal line (TVD) (*P* = 0.010), and area ratio of lesion to local epiphysis (lesion/growth plate) (*P* = 0.015). The MSTS93 (Revised Musculoskeletal Tumor Society Rating Scale 93) and SF-36 (Medical Outcomes Study 36-Item Short-Form Health Survey) had been significantly improved (*P* < 0.01).

**Conclusion:**

Managing of chondroblastoma located in open epiphyseal plate of a long bone with meticulous curettage, inactivity, and bone grafts can control tumor progression and recurrence effectively. Meanwhile, early detection and prompt surgical treatment intervention, which reduced significantly the tumor to influence limb growth and development, get encouraging limb function.

**Trial registration:**

This is a retrospective study, which was not registered in any trial registry.

## Background

Chondroblastoma is a rare benign bone tumor with an incidence of 9% in benign bone tumors [1]. Chondroblastoma commonly occurs in 10~25-year-old person. Previous studies revealed that it originated from chondroblasts, and entity was classified as a benign chondroblastoma of bone [2–5]. The chondroblastoma is typically located in the epiphysis of a long bone and less often in the apophysis [6–9]. Due to active and aggressive characteristics, chondroblastoma invade and destroy regularly adjacent tissues as tumor progression [6–9]. Meanwhile, knee, hip, and shoulder joints frequently are involved [9]. Common presenting symptoms include rest or activity pain and local tenderness, followed by swelling and limited motion of neighboring joints [9]. In serious cases, neighboring nerves and vascular joints are compressed and invaded. Thus, early detection and prompt treatment intervention are significant.

Surgery is recommended for treatment with chondroblastoma mainly [1, 9, 10]. The gold standard for surgical treatment is accurate and meticulous intralesional curettage with or without local adjuvant therapy followed by bone grafting [10]. The rate of recurrence after those procedures has been reported between 10 and 36% [3, 6, 7]. Some complications can be observed after surgical therapy due to involvement of the epiphysis, such as limb-length discrepancy and articular deformity with a frequency of 7–50% [9, 10].

To our knowledge, there were rare study series exclusively focusing on limb length of chondroblastoma after surgery in young patients with open epiphyseal growth plate [9, 10]. Epidemiologic characteristics and predictors of involved limb length of chondroblastoma during growth were not known. The objectives of the present retrospective study were to observe the effects of removed epiphyseal chondroblastoma during growth in 18 patients who were children and adolescents and to identify what factors might influence limb growth and development after surgical treatment.

## Methods

### Inclusion criteria

All patients with open epiphyseal growth plate of long bone chondroblastoma were accepted during March 2004 to October 2010 in our center. The chondroblastoma was confirmed by pathology. All patients received the first tumor resection. Operation agreement and rehabilitation protocol were signed.

### Exclusion criteria

The patient with closed epiphyseal plate was excluded. Trauma and pathological fracture of epiphyseal plate was not involved. Patients who suffered from poliomyelitis, congenital dementia, cretinism, cerebral palsy, and cartilage malnutrition were not unaccepted.

### Clinical data

We performed a retrospective study of 18 children and adolescents with chondroblastoma in our hospital from March 2004 to October 2010. The diagnosis was based on recognized image data and histological criteria including intraoperative frozen pathological and postoperative paraffin tissue biopsy. The general information is described in Table 1. All operations were performed in West China Hospital, Sichuan University by two senior surgeons (Drs. Chongqi Tu and Hong Duan). This study has been approved by the Ethics committee at West China Hospital of Sichuan University.

**Table 1:** Details of 18 patients

No	Age (years)	Sex	Location	Tumor activity	Tumor size (mm^3^)	TGD (mm)	TVD (mm)	Focus/growth plate	Follow-up (years)	Shorten (mm)
1	11	F	Proximal tibia	Active	35 × 50 × 50	50	− 20	25/50(50%)	9	30
2	13	M	Proximal tibia	Aggressive	30 × 40 × 55	55	3	15/40(38%)	7	28
3	11	F	Proximal tibia	Active	10 × 10 × 13	13	4	12/52(23%)	8.4	14
4	12	F	Distal femur	Active	20 × 23 × 26	26	3	15/55(27%)	8.5	14
5	14	M	Proximal humerus	Active	40 × 40 × 50	40	0	40/50(80%)	8.3	15
6	11	F	Distal femur	Active	15 × 15 × 20	15	10	13/53(25%)	10	12
7	14	M	Proximal tibia	Active	10 × 20 × 25	20	3	20/62(32%)	7	18
8	10	M	Distal femur	Aggressive	24 × 26 × 28	28	− 4	26/68(38%)	10	23
9	10	M	Proximal humerus	Aggressive	35 × 40 × 50	50	− 20	25/45(56%)	6.8	24
10	15	M	Proximal tibia	Active	35 × 40 × 50	50	− 15	25/50(50%)	9.5	20
11	8	F	Proximal tibia	Active	20 × 30 × 35	35	− 22	23/50(46%)	11.5	30
12	9	M	Proximal humerus	Aggressive	35 × 35 × 40	40	− 15	9/40(23%)	8.4	24
13	12	F	Proximal femur	Active	20 × 25 × 30	30	− 5	10/43(23%)	7.5	13
14	13	M	Proximal femur	Active	25 × 25 × 28	28	2	12/46(26%)	9.7	14
15	11	M	Proximal humerus	Active	20 × 25 × 35	20	− 7	20/50(40%)	7	15
16	14	M	Proximal humerus	Active	10 × 15 × 23	23	− 4	15/35(43%)	7.8	19
17	13	M	Proximal tibia	Active	10 × 12 × 14	14	0	7/35(20%)	5.5	1.5
18	9	F	Proximal humerus	Latent	10 × 10 × 12	12	5	9/37(24%)	5	18

The negative value indicates that the lesion involves epiphyseal plate; focus/growth plate (%), the largest area of the focus/lesion epiphyseal plate area

*Abbreviations: TGD* the greatest dimension of lesion, *TVD* the vertical dimension between edge of lesion and epiphyseal line

All patients underwent chest radiography and radionuclide bone imaging on presentation as a screening procedure for metastatic disease, and no metastatic disease occurred. The status of adjacent epiphyseal plate was determined by radiographs. An epiphyseal plate was classified as open if a wide and clearly defined radiolucency was apparent. Meanwhile, an epiphyseal plate was closed if an epiphyseal scar was present [10]. The lesion activity was classified as latent, active, and aggressive using of the system of Patrick C.et al. [11]. All 18 patients in our research had open epiphyseal plate according to this criterion.

### Measuring methods

**Fig. 1:**
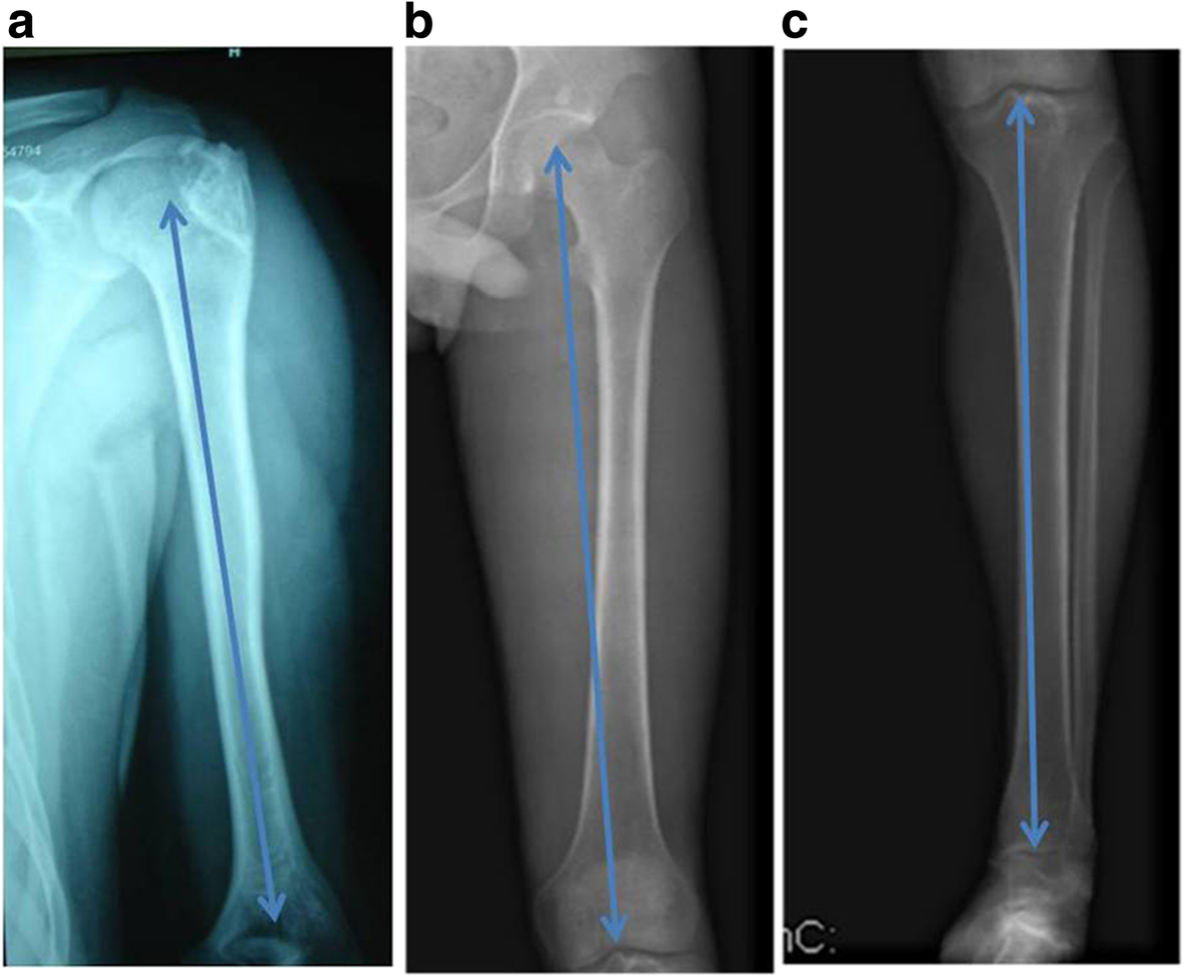
**a**–**c** The method of measuring length of the bone (the arrow straight line shows), humerus, from the humeral head midpoint to the midpoint of the medial and lateral condyle. The femur, from the midpoint of the femoral head to the midpoint of the medial and lateral condyle. The tibia, from the tibial plateau midpoint to within the lateral midpoint of the distal tibia

**Fig. 2:**
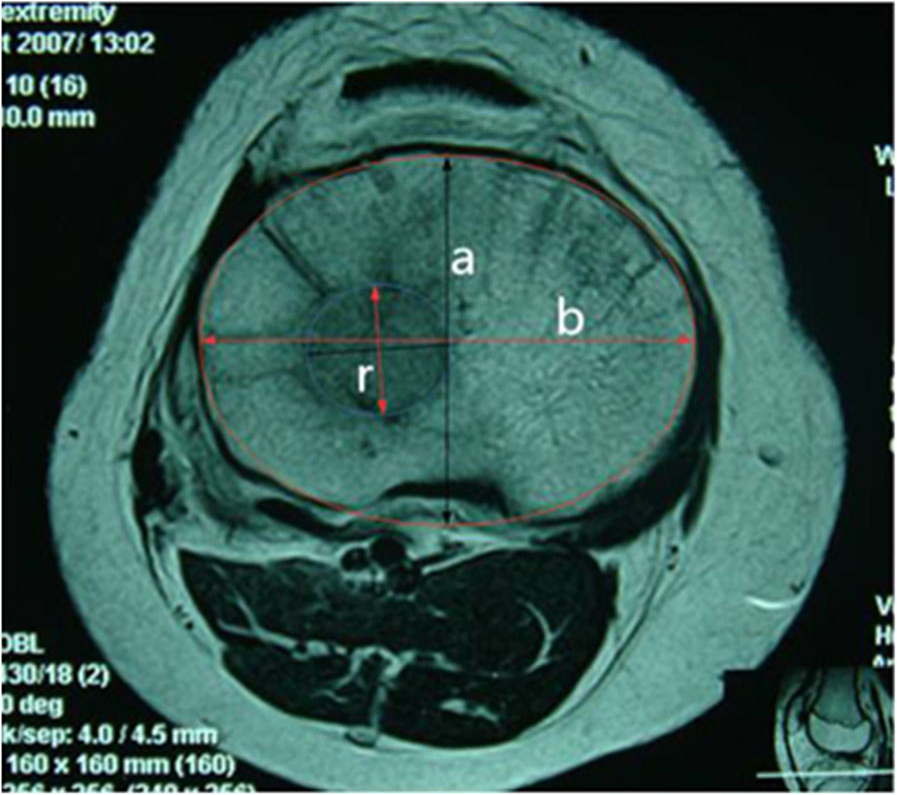
The method of measuring the lesion epiphyseal plate. r is the radius of the lesion, and a and b are minor and major axes of epiphyseal plate. Focus/epiphyseal plate = πr^2^/πab

**Fig. 3:**
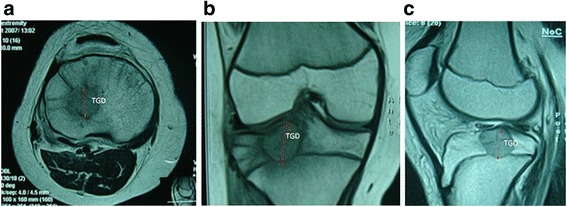
TGD as the greatest dimension of the lesion was measured according to MRI in the crown, axial, or sagittal plane

**Fig. 4:**
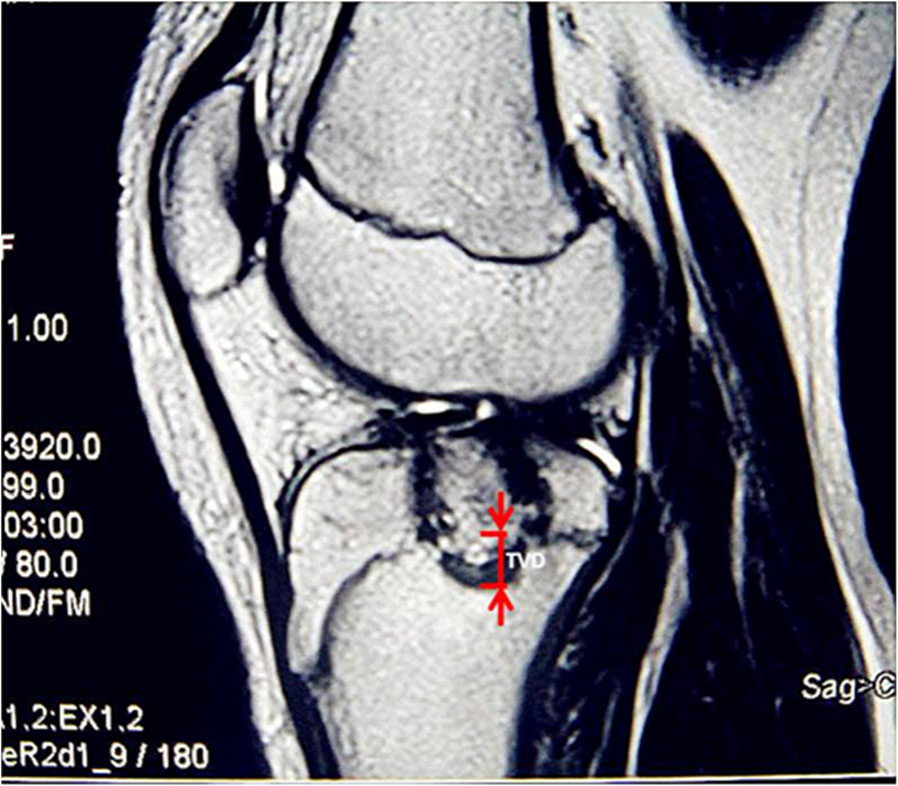
TVD, the vertical dimension between the edge of lesion and epiphyseal line, was from the lower edge of the lesion to the epiphyseal line in the vertical plane of the sagittal or crown MRI plane. TVD is a negative data when the lesion is crossing the epiphyseal line

We measured the length of each long bone according to X-ray on computer (Syngo version V35; Siemens Medical Systems, Erlangen, Germany) (Fig. 1). All lesions were nearly circular in the coronal plane in CT (computer tomography) or MRI (magnetic resonance imaging). Therefore, we measured the diameter of the biggest circular as 2r (radius of the lesion) and the minor and major axes of the oval as 2a (major axes) and 2b (major axes). Focus/epiphyseal plate as the area proportion of the circular and oval can be calculated from the following formula (focus/epiphyseal plate = πr^2^/πab, Fig. 2). The greatest dimension of the lesion (TGD) was measured according to MRI in the axial, sagittal, or crown plane (Fig. 3). The vertical dimension between the edge of lesion and epiphyseal line (TVD) was from the edge of the lesion reverse adjacent joint to the epiphyseal line in vertical plane of sagittal or crown MRI plane. The epiphyseal line was the flag line. The negative value indicated that the lesion damaged the epiphyseal line to involve the metaphyseal (Fig. 4).

### Surgical therapy

Firstly, all patients have undergone meticulous intralesional curettage from the local cortical window and polished tumor cavity around the lesion edge, 2–5 mm by bone drill (2 mm of latent lesion, 3 mm of active, 5 mm of aggressive, Table 1). Secondly, tumor cavity was inactivated by 95% alcohol for 15 min. Then, we used an electrotome to burn cavity in difficult-to-reach areas and saline solution to wash cavity repeatedly. Bone grafting was performed as follows. Allograft implantation was used in 13 patients, autologous iliac bone in 2 patients, and artificial bone (Medtronic, Inc., USA) in 5 patients. With the final processing, allogeneic bone block covered the cortical window, fixed with absorbable screw in seven patients and steel plate in a boy. There was no en bloc resection in this group. Patients were encouraged to perform a rehabilitation exercise with no weight bearing on the second day following surgery, such as joint mobilization and muscle strength training. In the sixth week, patients started partial weight bearing and full-weight bearing at the third month following surgery. At 6 month after surgery, all patients qualified for social work and sports activity.

### Follow-up observations

The follow-up occurred at 1, 2, 3, 6, 9, 12, and every 6 months thereafter. Imaging studies were focused on tumor recurrence and lesion limb growth and development. Local recurrence of the tumor was suspected if patient had persistent pain after surgery. We would carry out examination or MRI to exclude the pain from meniscus, cartilage, or soft tissue. Enlargement of the tumor on imaging studies, or bone marrow edema and cortical destruction on MRI, were thought to be signs of recurrence. Every 6 months after surgery, we scheduled a CT scan to master the status of bone grafting. The VAS scores, ISOLS grade, MSTS scores, and SF-36 scores were used to evaluate surgery effects. A comprehensive psychological intervention or treatment was performed in each follow-up. To explore the effect of surgical treatment on limb growth and development, we counted the shorten length of the lesion limb by X-ray examination and analyzed the relation between the shorten length with tumor activity, focus/epiphyseal plate, TGD, and TVD.

### Statistical analysis

SPSS 19.0 (IBM Corporation, Armonk, NY, USA) software was used. The values are presented as mean ± standard deviation (SD). Rank correlation was used to determine the relationship between the shorten length with tumor activity, focus/epiphyseal plate, TGD, and TVD. *r*_s_ was Spearman’s rank correlation coefficient. Paired samples *t* test was used to determine differences of VAS, MSTS93, and SF-36 between preoperation and the last follow-up. A *p* value of less than 0.05 was considered significant.

## Results

**Fig. 5:**
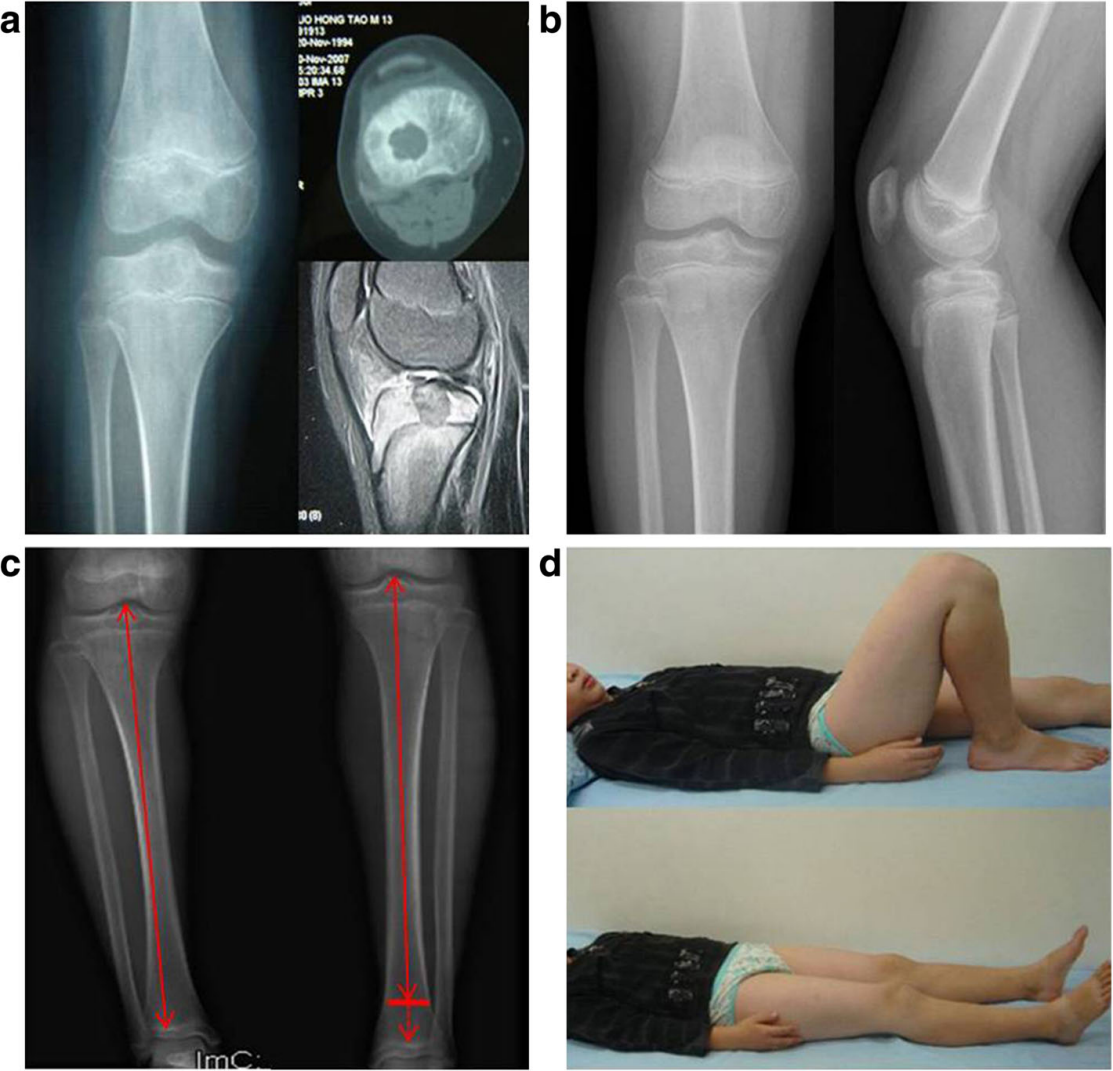
**a** Preoperative radiographs of a 11-year-old girl. The radiolucent lesion (arrows) and bone cortical erosion in CT and MRI were visible in the proximal tibia. **b** and **c** were the X-rays of 3 and 5 years in the course of follow-up. We can see that the density of the tumor cavity become more and more high in the X-ray postoperative. Eight years later, the troubled limb was shorten by 14 mm compared with the healthy limb and mild varus deformity but had a satisfied function of the knee (**d**)

**Fig. 6:**
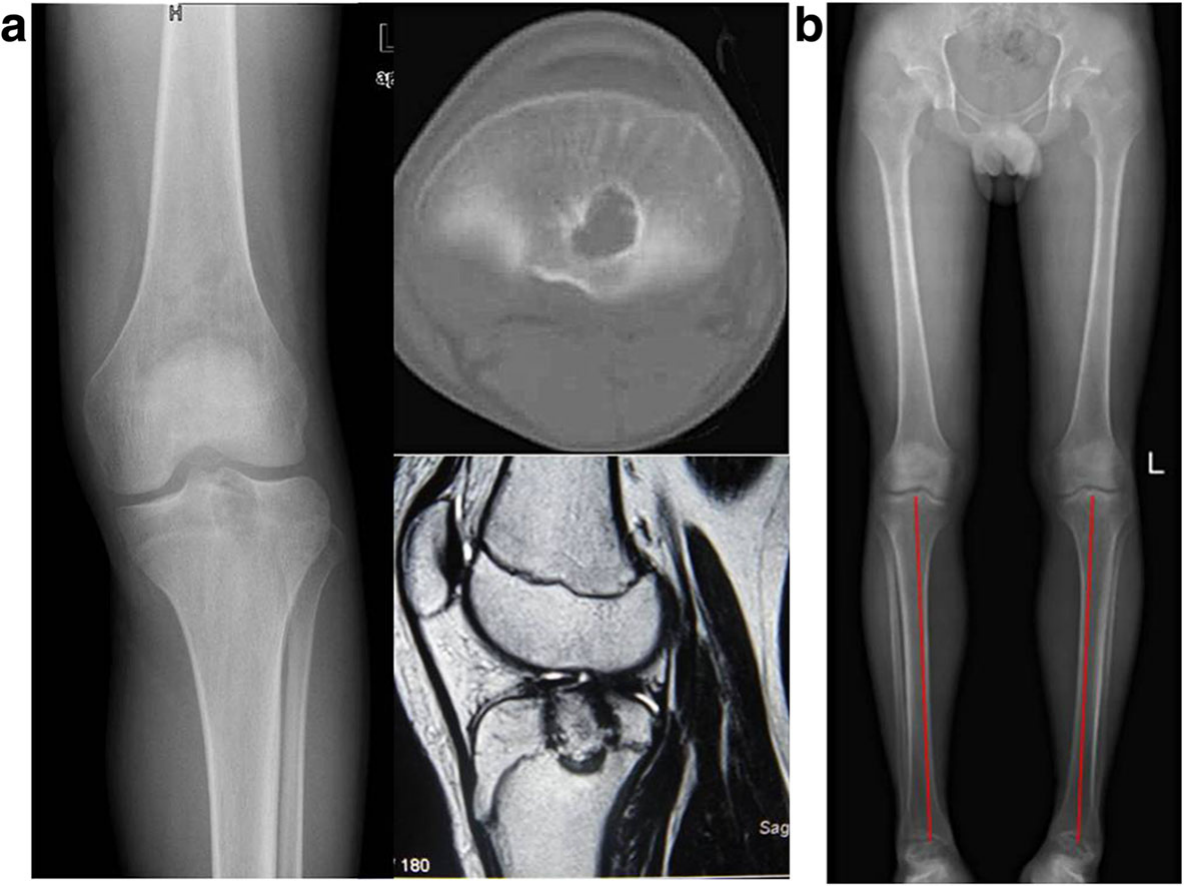
A 13-year-old boy’s X-ray, CT, and MRI (**a**) showed the lesion located at the proximal tibia. X-ray at 5.5 years postoperative told us that the grafting bone had been incorporated with the host bone and that the troubled limb shorten by 1.5 mm (**b**) compared with the healthy limb

All patients were received follow-up with the median of 8.2 ± 1.7 years (5–11.5 years). The outcomes were summarized in Table 1. All wounds healed to grade A. No postoperative infections, delayed deep infection, nonspecific inflammation, rejection, allergies, hypersensitivity, and fractures were encountered. There was no evidence of local recurrence and distance metastasis in all cases. The patients’ pain was completely resolved following surgery. There was no traumatic arthritis, joint collapse, or chronic joint pain in long-term follow-up. The ISOLS (International Society of Limb Salvage) functional grade was 28.67 ± 1.24 on average at the last follow-up. The function of MSTS93 and SF-36 have been significantly improved (*P* < 0.01) (Table 2, Figs. 5 and 6). Eighteen patients have obtained excellent range of motion. Just an 11-year-old girl was observed with 5° valgus deformity in the left tibia at 1-year anniversary. There was radiographic evidence of bone grafts completely incorporated postoperative 12 to 18 months.

**Table 2:** The follow-up outcomes (mean ± SD)

	VAS	MSTS	ISOLS	SF-36				
				GH	PF	VT	RE	Soc
Preoperation	6.33 ± 1.97	23.67 ± 1.46	None	58.33 ± 11.11	58.61 ± 11.48	51.39 ± 15.79	55.56 ± 10.13	46.11 ± 14.71
Last follow-up	0.17 ± 0.38*	28.56 ± 1.34*	28.67 ± 1.24	91.11 ± 4.39*	91.67 ± 3.43*	88.61 ± 5.37*	90.28 ± 3.63*	89.17 ± 5.22*

*Abbreviations: VAS* visual analog scale, *MSTS93* Revised Musculoskeletal Tumor Society Rating Scale, *SF-36* Medical Outcomes Study 36-Item Short-Form Health Survey, *ISOLS* International Society of Limb Salvage, *GH* general health, *PF* physical function, *VT* vitality, *RE* role emotional, Soc, social function

*Compared with preoperative, the differences were significant (*P* < 0.01)

**Fig. 7:**
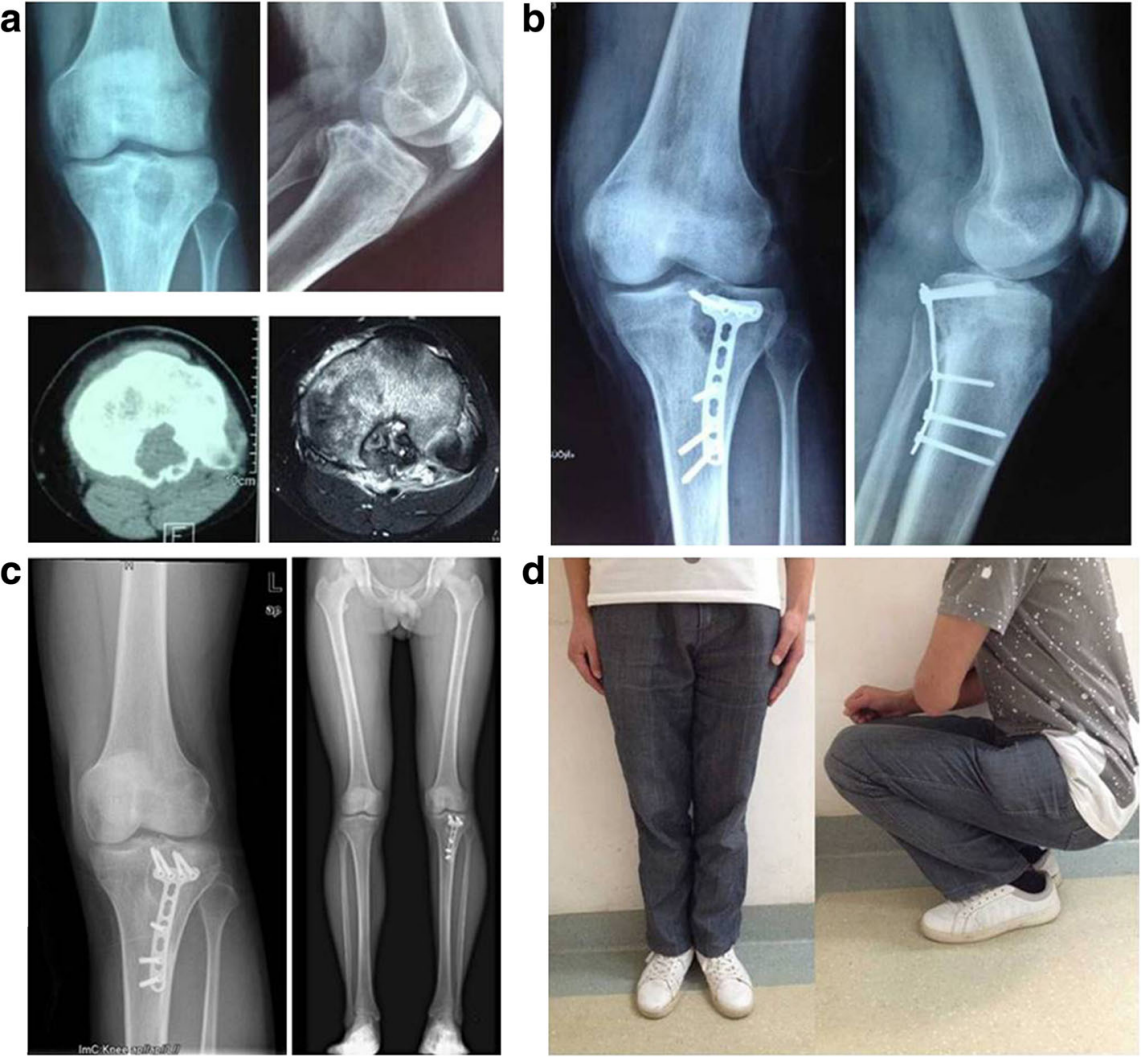
A 15-year-old boy’s X-ray , CT , MRI (**a**)showed the lesion located at posterior median of the proximal tibia. X-ray at 1 year(**b**) and 9.5 years(**c**) postoperative told us that the grafting bone had been gradually incorporated with the host bone and that the troubled limb shortened 20 mm compared with the healthy limb(**d**). The patient acquired a satisfactory function outcome and could run 3 min 43 s in 1-km race

The length of lesion limbs were shortened 18.47 ± 7.22 mm (1.5–30 mm) compared with non-surgery limb (Table 1). The shorten length have no obvious relativity with tumor activity (*P* = 0.061), but obvious relativity focus/epiphyseal plate (*P* = 0.015), TGD (*P* = 0.003), and TVD (*P* = 0.010) (Table 3). In relation to the shorten length and TVD, *r*_s_ = − 0.591 was a negative correlation, which exposed that the bigger the value was, the lesser effective to the length shortening. In other words, the lesion crossing the epiphyseal line more led to more severe limb shortening (Fig. 7).

**Table 3:** Statistical data on 18 patients

	*r*_s_	*P*
Tumor activity	0.450	0.061
TGD (mm)	0.665	0.003
TVD (mm)	− 0.591	0.010
Focus/growth plate	0.565	0.015

The negative value indicates that the lesion involves the epiphyseal plate; focus/growth plate, proportion between the largest area of the focus lesion in the horizontal and the epiphyseal plate. *r*_s_ was Spearman’s rank correlation coefficient

*Abbreviations: TGD* the greatest dimension of the lesion, *TVD* the vertical dimension between the edge of lesion and epiphyseal line

## Discussion

Chondroblastoma is a benign tumor and mostly originates in an epiphyseal plate of long bone, which primarily occurs in children and adolescents. Previous studies reported first-line treatment should be lesion curettage [1, 9, 12, 13]. Local recurrence rate of lesion curettage was 10~35%. Risk factors of recurrence include location, young age, inadequate surgery, and biologic aggressiveness of tumor [12, 13]. Schreuder et al. reported that surgical technique might play the most important role in chondroblasoma recurrence [12]. Some reports showed that simple curettage was associated with higher recurrence rate because of too much worry about damaging an open epiphyseal plate [12, 13]. In this study, we preferred to use a bone drill to polish tumor cavity. With our experience, 2 mm of latent lesion, 3 mm of active, and 3–5 mm of aggressive were appropriate to remove residual tumor cells. We also took chemical (95% alcohol) and thermal (electrotome) methods to inactivate tumor cavity. However, in areas near or crossing the epiphyseal line, we would open a small cortical window to remove tumor, which could minimize the epiphyseal line being injured. Besides, using a bone drill was relatively conservative, while chemical and thermal methods were more radical. Thus, those pre-processing techniques could prevent tumor recurrence drastically and reduce epiphyseal plate damage.

The epiphyseal plate is located between the epiphysis and metaphysis of long bones, which has complex anatomy with the following cellular layers: reserve zone, proliferative zone, and hypertrophic zone. It regulates endochondral maturation, degeneration, and calcification [14, 15]. The reserve zone is called the germinal or stem cell zone, which contains resting chondrocytes. The combination of chondrocyte proliferation, the enlargement of maturing chondrocytes in the hypertrophic zone, and the production of ECM (extracellular matrix) are the major contributors to longitudinal bone growth [16]. Tumor curettage may hurt the epiphyseal plate. However, the epiphyseal plate has limited ability to repair. Furthermore, vascular of metaphysis invaded into the broken epiphyseal plate and formed a fiber vascular bridge. Finally, a bone bridge was formed with a large number of calcium salt depositions, which is lacking of longitudinal growth ability, leading to limb shortening and angular deformity [17].

Previous studies have found that resistance of long bones to growth and development is related to the range of injured epiphyseal plate [17, 18]. When the injured percentage of the central area is more than 7%, the bone bridge formed and stretched the whole epiphyseal plate. Meanwhile, the eccentric lesion percentage could be more than 9%, which caused obvious resistance [19, 20]. In our study, all intralesion curettage led to epiphyseal plate injury, and many lesion areas were more than 10%, one was up to 50%. By follow-up, it resulted in limb shortening and deformity differentially as the body grows. Our outcome showed that focus/epiphyseal plate, TGD, and TVD were the key factors in a limb-growing capability disorder. In relation to shortened length and TVD, the lesion crossing the epiphyseal line more led to more severe limb shortening and deformity. In our series, the length of troubled limb were shorter 18.47 ± 7.22 mm (1.5–30 mm) compared with the healthy limb on 5 upper limbs and 13 lower limbs. Thirteen patients whose lesions were located in the lower limbs had no symptoms on walking because of a pelvic compensator, and the other five patients’ shoulder function was satisfied at the last follow-up.

Limb length discrepancy and angular deformity may not necessarily lead to clinical problems during childhood and puberty, but psychosocial problems may occur. Social withdrawal, practical problems relating to clothing and shoes, fearing about future compatible partners, and career planning would suffer [21–23]. Lower limb length discrepancy would lead to posture deformation, gait asymmetry, low back pain, and discopathy [21, 22]. Thus, in addition to clinical problem improvement, it was necessary to make a comprehensive psychological intervening or treatment to guide patient’s healthy and joyful growth. As we have known, leg length discrepancy < 2 cm is a static disorder. Leg length discrepancy > 3 cm causes distinct gait and posture disorders—the bigger the difference the greater and more distinct the disorders are [22]. In our study, all the patients underwent positive psychological intervention and clinical symptoms treatment after surgery. All patients’ function of VAS, MSTS93, and SF-36 have been significantly improved (*P* < 0.01). They all joined in social activities in adulthood as farmer, construction worker, college students, civil servants, and teachers, and so on.

## Conclusion

Managing of chondroblastoma located in an open epiphyseal plate of a long bone with meticulous curettage, inactivity, and bone grafts can control tumor progression and recurrence effectively. Meanwhile, early detection and prompt surgical treatment intervention, which reduced significantly the tumor to influence limb growth and development, get encouraging limb function.

### Limitation

First, as a retrospective study, we had no control group to assess the superiority of our study compared with different tumor management. This was a retrospective review and is, therefore, limited by the heterogeneity of the available data and follow-up. Second, the sample size was few. We needed more cases and time to observe local recurrence, distant metastasis, limb discrepancy, and function. Furthermore, because of few cases in our study, we could not observe more factors, which might influence limb growth and development after surgical treatment, such as age and gender.
